# Multi-System Deconditioning in 3-Day Dry Immersion without Daily Raise

**DOI:** 10.3389/fphys.2017.00799

**Published:** 2017-10-13

**Authors:** Steven De Abreu, Liubov Amirova, Ronan Murphy, Robert Wallace, Laura Twomey, Guillemette Gauquelin-Koch, Veronique Raverot, Françoise Larcher, Marc-Antoine Custaud, Nastassia Navasiolava

**Affiliations:** ^1^Mitovasc, UMR Institut National de la Santé et de la Recherche Médicale 1083, Centre National de la Recherche Scientifique 6015, Université d'Angers, Angers, France; ^2^Russian Federation State Research Center, Institute of Biomedical Problems, Russian Academy of Sciences, Moscow, Russia; ^3^Center for Preventive Medicine, School of Health and Human Performance, Dublin City University, Dublin, Ireland; ^4^Centre National d'Etudes Spatiales, Paris, France; ^5^Hospices Civils de Lyon, Lyon, France; ^6^Laboratoire de Biochimie, Centre Hospitalier Universitaire d'Angers, Angers, France; ^7^Centre de Recherche Clinique, Centre Hospitalier Universitaire d'Angers, Angers, France

**Keywords:** modeled weightlessness, physical inactivity, supportlessness, cardiovascular deconditioning, glucose intolerance, muscle tone, day-night variations, kaliuresis

## Abstract

Dry immersion (DI) is a Russian-developed, ground-based model to study the physiological effects of microgravity. It accurately reproduces environmental conditions of weightlessness, such as enhanced physical inactivity, suppression of hydrostatic pressure and supportlessness. **We aimed** to study the integrative physiological responses to a 3-day strict DI protocol in 12 healthy men, and to assess the extent of multi-system deconditioning. We recorded general clinical data, biological data and evaluated body fluid changes. Cardiovascular deconditioning was evaluated using orthostatic tolerance tests (Lower Body Negative Pressure + tilt and progressive tilt). Metabolic state was tested with oral glucose tolerance test. Muscular deconditioning was assessed via muscle tone measurement.

**Results:** Orthostatic tolerance time dropped from 27 ± 1 to 9 ± 2 min after DI. Significant impairment in glucose tolerance was observed. Net insulin response increased by 72 ± 23% on the third day of DI compared to baseline. Global leg muscle tone was approximately 10% reduced under immersion. Day-night changes in temperature, heart rate and blood pressure were preserved on the third day of DI. Day-night variations of urinary K^+^ diminished, beginning at the second day of immersion, while 24-h K^+^ excretion remained stable throughout. Urinary cortisol and melatonin metabolite increased with DI, although within normal limits. A positive correlation was observed between lumbar pain intensity, estimated on the second day of DI, and mean 24-h urinary cortisol under DI. **In conclusion**, DI represents an accurate and rapid model of gravitational deconditioning. The extent of glucose tolerance impairment may be linked to constant enhanced muscle inactivity. Muscle tone reduction may reflect the reaction of postural muscles to withdrawal of support. Relatively modest increases in cortisol suggest that DI induces a moderate stress effect. **In prospect**, this advanced ground-based model is extremely suited to test countermeasures for microgravity-induced deconditioning and physical inactivity-related pathologies.

## Introduction

Spaceflight induces physiological multi-system deconditioning which may impact astronauts efficiency and create difficulties upon their return to normal gravity (Nicogossian et al., 1993). Understanding the underlying mechanisms of this process and enhancement of countermeasures remains a challenge and major priority for manned space programs. Moreover, resultant data and experience may be used to resolve common earth-based chronic healthcare problems related to increased physical inactivity, for example post-stroke patients, bedridden, paralyzed or immobilized subjects, sedentary people, aging etc. Experimental opportunities during actual spaceflight being limited, the appeal of ground-based simulations is obvious and paramount (Pavy-Le Traon et al., 2007). Dry immersion (DI) is one such prolonged microgravity model. It accurately reproduces most physiological effects of microgravity, including centralization of body fluids and hypokinesia (Kozlovskaia, 2008; Navasiolava et al., 2011a; Watenpaugh, 2016). The benefit of DI, compared to more widely-known and traditional head-down bed rest (HDBR) technique, is support unloading (“supportlessness”), a state akin to weightlessness, with water hydrostatic pressure equally distributed over the body surface, providing conditions similar to complete lack of structural support (Grigor'ev et al., 2004; Navasiolava et al., 2011a). DI promotes rapid gravitational deconditioning, exceeding for some systems (i.e. for neuromuscular system) the deconditioning induced by spaceflight itself (Navasiolava et al., 2011a). However, this DI method, developed and widely used in Russia, is not yet routine elsewhere. Moreover, all DI experiments performed until now included a short daily raise for personal hygiene procedures and weighing (Navasiolava et al., 2011a). Importantly, literature shows that this type of short daily orthostatic stimulation could act as a countermeasure (Greenleaf, 1984; Vernikos et al., 1996). Therefore, in order to eliminate this aberration, our novel DI protocol did not permit subjects to rise at all for 3 days, and a −6° head down position was maintained when the subjects were out of water, as is observed in strict bedrest protocols. Hence, this study is the first DI protocol specially conceived to assess integrative aspects of “strict” DI impact.

We aimed to study integrative response to 3-day strict DI, and to assess the extent of multi-system deconditioning with regard to cardiovascular, metabolic, muscular system, day-night changes in renal excretion, and adaptive capacities/ stress effect.

## Materials and methods

A total of twelve healthy non-athletic men aged 26 to 39 year. o. (age 32 ± 1.4 yr, weight 75 ± 2 kg, height 178 ± 2 cm, BMI 23.6 ± 0.4 kg/m^2^, maximal oxygen uptake ${\dot{\text{V}}\text{O}}_{2}$max 39 ± 1.1 mL/min/kg) were included in the study. Subjects had no history of cardiovascular or other chronic diseases, and were not taking medication prior to the experiment. All subjects were informed about the experimental procedures and gave their written consent. The experimental protocol conformed to the standards set by the Declaration of Helsinki and was approved by the local Ethic Committee (CPP Sud-Ouest Outre-Mer I, France) and French Health Authorities (n° ID RCB: 2014-A 00904-43).

### General protocol

**Figure 1:**
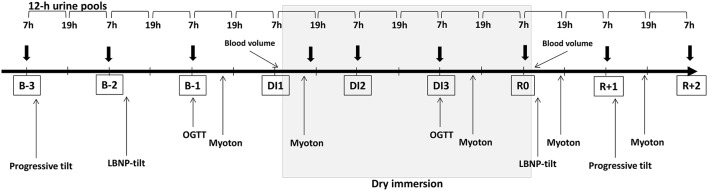
Global protocol timeline. Thick arrows stand for blood sampling. OGTT, Oral Glucose Tolerance Test; LBNP, Lower Body Negative Pressure; B-3, B-2, B-1, days before dry immersion; DI1, DI2, DI3, first, second and third days of dry immersion. R0, R+1, R+2–days after dry immersion.

The study was conducted at the MEDES space clinic, Toulouse, France. The experimental protocol lasted for 8 days: 3 days of ambulatory baseline measurement before immersion (B-3, B-2, B-1), 3 days (72 h) of dry immersion (DI1, DI2, DI3) and 2 days of ambulatory recovery (R0, R+1). Two subjects in two separate baths underwent DI simultaneously. Thermoneutral water temperature (32.5–33.5°C) was continuously maintained. Light-off period was set at 23:00–07:00. General discomfort and lumbar pain under immersion were evaluated using a visual analog scale. Daily hygiene, weighing and some specific measurements required extraction from the bath. During these short out-of-bath periods, subjects maintained the −6° head-down position. Total out-of-bath supine time for the 72 h of immersion was 4.7 ± 0.16 h. Otherwise, during DI, subjects remained immersed in a supine position for all activities and were continuously observed by video monitoring. Blood pressure, heart rate (HR) and tympanic body temperature were measured twice daily at 07:00 and 19:00. Body weight was measured daily at 07:00. Onset and end of immersion both occurred at 09:00, therefore morning measurements on DI1 were performed before immersion, and on day R0-still under immersion. Before, during and after DI, water intake was *ad libitum* (measured), and diet was the same for all participants and standardized to body weight in energy and nutrients. Daily caloric intake was approximately 2,820 kcal for baseline and recovery and 2,270 kcal for the immersion period. Daily intake for sodium and potassium was approximately 3–4 g. Daily nutrition is detailed in supplementary data (Table S1). The timeline schematic of the global protocol is outlined in Figure 1.

This 3-day DI allowed for several protocols on different domains performed by 8 research groups. Some studies have already been published to-date (Treffel et al., 2016a,b; Arbeille et al., 2017; Demangel et al., 2017).

### Lower body negative pressure (LBNP)-tilt test

Tilt testing with combined lower body negative pressure (LBNP) was chosen as the accepted standard for measuring orthostatic tolerance (Protheroe et al., 2013). The measurement was conducted in the morning in a temperature-controlled room (22 ± 0.6°C) at baseline on B-2 and immediately following DI on R0 (first rising after DI). The subject remained supine for 20 min, after which supine data were recorded for 5 min. The tilt-table was then rotated to 80° for 15 min. After that, LBNP was applied with steps of −10 mmHg every 3 min. The test was stopped at LBNP −60 mmHg or earlier upon appearance of pre-syncopal signs, request to stop, systolic blood pressure ≤ 80 mmHg, HR < 50 bpm or > 170 bpm.

During the LBNP-tilt test, finger blood pressure (Nexfin, BMeye, USA) and standard ECG (Biopac, ECG 100C, USA) were recorded continuously. Orthostatic tolerance time, heart rate (HR), blood pressure (systolic, diastolic), stroke volume (SV), total peripheral resistance were estimated. Stroke volume and total peripheral resistance were evaluated from the blood pressure wave using the modelflow® method (Beatscope® software, TNO, the Netherlands). The state of autonomic nervous system was estimated via power spectrum analysis of heart rate variability. Sympathetic index, an indicator of sympatho-vagal balance, was calculated as the ratio of low-to-high frequency spectral power. Spontaneous baroreflex sensitivity was estimated using online software (televasc.fr).

### Progressive tilt test

Progressive tilt was used to progressively stimulate the cardiovascular system (volume receptors for low angles and baroreceptors for higher angles) and vestibular system. A head-up tilt test with progressive angles (0, 20, 45, and 80°) was performed at baseline on B-3 and on the second recovery day (R+1, 24 h following the completion of the DI protocol). After 10 min of supine rest recording, the subject was tilted at 20° (5 min), 45° (5 min) and 80° (5 min), and then returned to the horizontal position.

During the progressive tilt test, HR, systolic and diastolic blood pressure, total peripheral resistance, stroke volume, sympathetic index, spontaneous baroreflex sensitivity were calculated and recorded.

### Blood studies

Antecubital venous blood samples were collected before (B-3, B-2, B-1), during (DI1-evening, DI2, DI3, R0) and after DI (R+1, R+2). Blood sampling was performed in the morning before breakfast, except for DI1 when it was done in the evening (10h of DI).

Plasma and serum samples were analyzed for electrolytes (Na+, K+, Cl−), glucose, proteins, albumin, urea, and creatinine concentrations, high-sensitivity CRP, insulin, leptin, triglycerides, total cholesterol, and HDL-cholesterol. LDL-cholesterol was calculated using the Friedewald formula. Homeostasis model assessment-insulin resistance index (HOMA-IR) was calculated as fasting insulin concentration (μU/mL) × fasting glucose concentration (mmol/L)/22.5.

Additionally, on B-3 and R+1 blood count, GGT, ALT, AST, alkaline phosphatase, total bilirubin and prothrombin time were assessed. Hb, Hct, renin, aldosterone and blood natriuretic peptide (BNP) were assessed on B-3, R0 and R+1.

### 75-g oral glucose tolerance test

Glucose tolerance tests were performed in the morning on B-1 and DI3 (48 h of immersion). Blood glucose and insulin were measured after an overnight fast before and 30, 60, 90, and 120 min after a 75-g glucose intake (consumption of glucose solution drink within 5 min).

The total area under the blood glucose curve (AUC-Glu) and insulin curve (AUC-Ins) during the 75-g oral glucose tolerance test were calculated using the trapezoidal rule.

### Urine sampling

Urine pools were collected over 12 h (07:00–19:00 pool for “day” and 19:00–07:00 pool for “night”) throughout the protocol. Light on/off periods were not taken into account for urine collection, therefore “night” pools included 4 h of light-on (07:00–23:00) and 8 h of light-off (23:00–07:00). Urine volume was measured, and aliquots stored at −80°C. Partial water balance, defined as the difference between consumed water and urine volume, was calculated. Urine samples were analyzed for electrolytes (Na^+^, K^+^), cortisol and 6-sulphatoxymelatonin (aMT6s, a hepatic metabolite of melatonin usually used as a proxy measure of melatonin level).

### Biochemical analyses

Active renin analysis was performed using a chemiluminescence immunoassay on the Liaison analyzer (DiaSorin). Plasma aldosterone was determined by a competition radioimmunoassay using a commercially available RIA kit (Immunotech, Beckman Coulter). Urinary cortisol and aMT6s assays were carried out by radioimmunoassay. All other variables from blood and urine samples were evaluated using the Architect c16,000 automated clinical chemistry analyzer (Abbott). Minimal detectable levels for aldosterone and BNP were 10 ng/L, for hs-CRP–0.1 mg/L. Results that were less than the minimal detectable level, were taken for half minimal value.

### Blood and plasma volume measurement

Blood volume and plasma volume were estimated using the optimized CO-rebreathing method (Schmidt and Prommer, 2005) in the morning before breakfast on DI1 just before the onset of immersion and on R0 immediately at the end of immersion, in supine position. Additionally, percent change in plasma volume on R0 and R+1 vs. B-3 was calculated using Hb and Hct count (Dill and Costill formula): DPV (%) = 100 × [HbB (1−0.01Hcti)]/ [Hbi (1−0.01HctB)] − 100, where HbB and HctB are baseline Hb and Hct levels, and Hbi and Hcti are Hb and Hct on days R0 and R+1, respectively.

### Muscle tone

Mechanical characteristics of muscles were determined using a hand-held myotonometer (MyotonPRO; Myoton Ltd, Estonia) before (B-1), 6 h following onset (DI1) and on day 3 of immersion (DI3), and in recovery period on R0 (6 h following the end of DI) and on R+1.

During the tests, the subject was out-of-water in a relaxed supine position (dorsal and ventral decubitus). The testing end of the myotonometer was kept perpendicular to the muscle's projection in the middle of the muscle belly, at the same muscle point throughout the study. The device was used in multiscan mode, where one measurement corresponded to the mean of 5 mechanical taps. Leg measurements were taken on the left *m. rectus femoris, m. tibialis anterior, m. gastrocnemius lateralis* and *m. soleus*. Trunk measurements were taken on the right and left *m. splenius, m. trapezius, m. longissimus cervicis, m. longissimus thoracis* and lumbar portion of *m. multifidus*. Since trunk data showed no difference between the right and left sides (all *p* > 0.05), results from right and left sides were averaged. For technical reasons, the data of 2 subjects (subjects C and D) have not been analyzed.

### Statistical analysis

The values are presented as mean ± SEM. Statistical analysis was performed with Prism6 GraphPad. Data were compared by a one-way and two-way repeated measures ANOVA followed by post hoc Bonferroni test. Relationships between data were examined using the Pearson correlation coefficient (r). Detailed correlations are given in supplemental data (Table S2).

## Results

### General data

**Figure 2:**
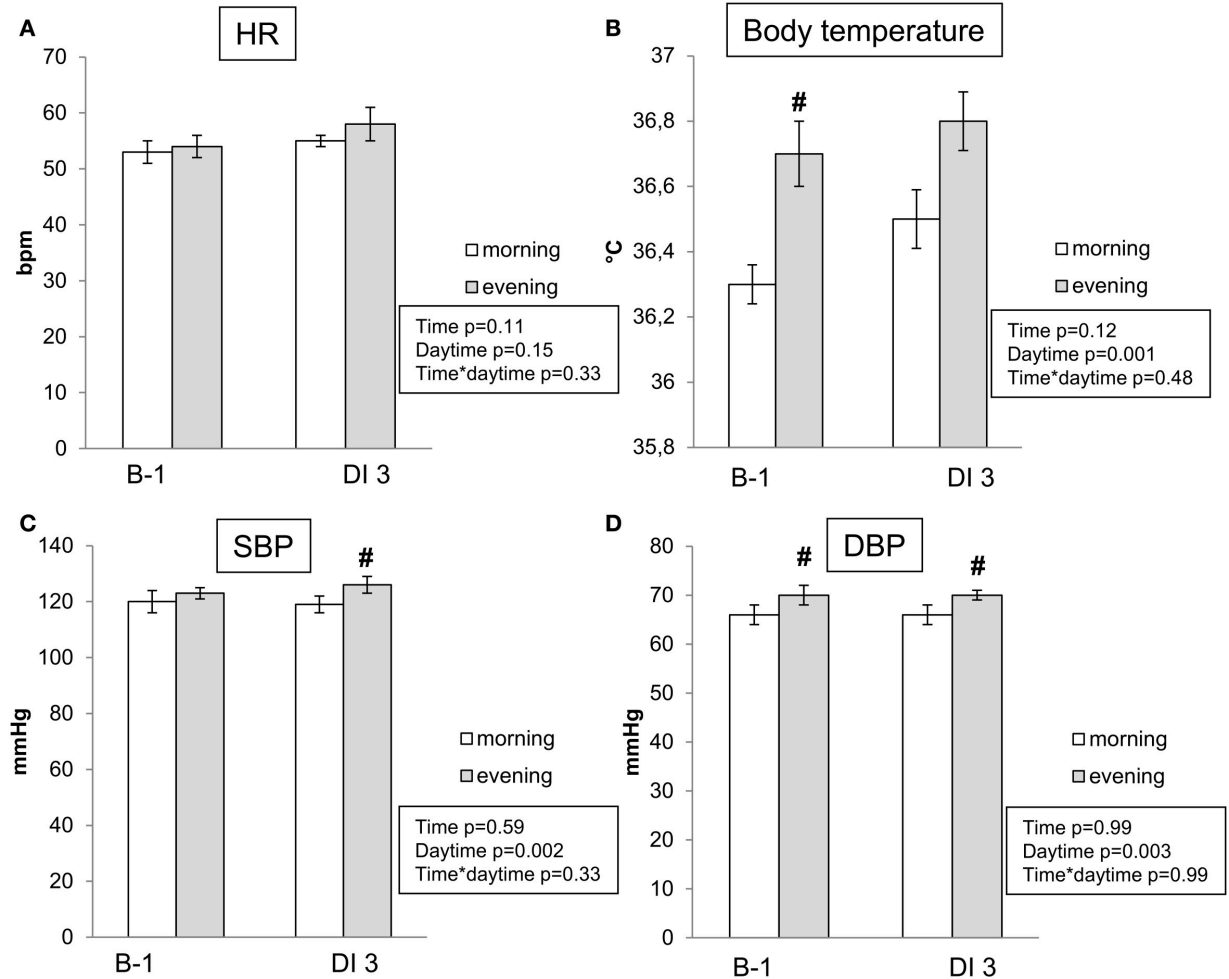
Morning and evening heart rate **(A)**, body temperature **(B)**, systolic **(C)**, and diastolic **(D)** blood pressure before DI and on the 3rd day of DI. Data are mean ± SEM. No significant difference on DI3 vs. B-1. #*p* ≤ 0.05 vs. Morning.

HR, blood pressure and body temperature remained within normal limits throughout the protocol. Baseline values of blood pressure and temperature were slightly higher in the evening. On the third day of immersion HR, blood pressure and body temperature did not differ significantly from B-1 baseline level (Figure 2). Body weight had decreased by 1–2 kg on the third day of immersion (Table 1).

**Table 1:** Body weight, water intake, diuresis, and partial water balance.

	**B-1**	**DI1**	**DI2**	**DI3**	**R0**	**R+1**	***p*-value (RM ANOVA)**
Morning weight, kg	75.4 ± 2.1	74.9 ± 2.1^*^	73.8 ± 2.0^*^	73.7 ± 2.0^*^	73.7 ± 2.0^*^	74.2 ± 2.1^*^	<0.001
Water intake, ml	2,975 ± 115	2,181 ± 138^*^	2,295 ± 184^*^	2,072 ± 93^*^	2,994 ± 135	3,069 ± 158	<0.001
Diuresis, ml	2,347 ± 157	2,343 ± 180	1,860 ± 150	1,478 ± 117^*^	1,356 ± 76^*^	1,763 ± 162^*^	<0.001
Partial water balance, ml	628 ± 74	–162 ± 134^*^	435 ± 88	594 ± 85	1,638 ± 114^*^	1,306 ± 97^*^	<0.001

Values are mean ± SEM;

* *p ≤ 0.05 vs. B-1*.

### Body fluids

#### Diuresis, water intake and partial water balance

Data are shown in Table 1. Water intake set *ad libitum* was decreased as anticipated on DI1, due to the known reset of water balance to lower level (Navasiolava et al., 2011c), but also on DI2 and DI3. This was likely due to discomfort during subject urination in the water tank, causing a voluntary reduction of water intake. Diuresis remained unchanged on DI1 despite a 30% reduction in water intake, therefore partial water balance decreased up to 700–800 mL and became negative on the first day of DI. On DI2 and DI3, water intake remained approximately 30% reduced, together with decrease in diuresis. At the recovery stage, water intake returned to baseline values, while diuresis remained diminished, with a compensatory increase in water balance.

#### Blood and plasma volume

Blood volume decreased by 11% on R0 (from 6.45 ± 0.20 L to 5.74 ± 0.17 L, *p* < 0.001), while total Hb mass diminished by 3% (from 887 ± 33 g to 861 ± 33 g, *p* = 0.009). In relation to plasma volume, the CO technique showed a 16 ± 2% decrease at DI3 compared to DI1, and the Dill and Costill estimation showed a 14 ± 2% decrease vs. B-3. Hence, there was no significant difference between these two types of measurements. There were no changes in plasma volume at R+1 compared to baseline. Pre-immersion plasma volume expressed in ml/kg correlated with initial ${\dot{\text{V}}\text{O}}_{2}$max (Pearson *r* = 0.67; *p* = 0.017), and percent reduction in plasma volume also correlated with initial ${\dot{\text{V}}\text{O}}_{2}$max (Pearson *r* = −0.76; *p* = 0.006), i.e., fitter subjects had greater relative plasma volume at baseline and greater hypovolemia under DI.

#### Volume-regulating hormones (renin-angiotensin-aldosterone system and BNP)

A 25% increase in renin was observed prior to the end of DI (morning of R0). Twenty-four hours after DI3, renin was expectedly increased by two-fold, and aldosterone by 3-fold. Brain Natriuretic Peptide (BNP) was below the minimum detectable level in all subjects on DI3 and R+1 compared to 8.3 ± 1.6 ng/L on B-3 before DI (Table 2).

**Table 2:** Blood assessment (chemistry, cardiovascular hormones, metabolic parameters, blood count).

**Variable**	**Baseline**	**DI1 10 h of DI**	**DI2 22 h of DI**	**DI3 46 h of DI**	**R0 70 h of DI**	**R+1 +22 h**	***p*-value (RM ANOVA)**
Sodium, mmol/L	137 ± 1.1	142 ± 0.8^*^	144 ± 1.1^*^	139 ± 1.0	139 ± 0.7	139 ± 0.5	<0.001
Chlorine, mmol/L	101 ± 0.7	105 ± 0.5^*^	105 ± 0.7^*^	102 ± 0.6	102 ± 0.3	102 ± 0.4	<0.001
Potassium, mmol/L	3.9 ± 0.04	4.0 ± 0.12	4.3 ± 0.09	4.2 ± 0.04^*^	4.2 ± 0.07	4.1 ± 0.06	0.03
Proteins, g/L	63 ± 1	70 ± 1^*^	72 ± 1^*^	71 ± 1^*^	70 ± 1^*^	67 ± 2	<0.001
Albumin, g/L	40 ± 0.5	43 ± 0.3^*^	44 ± 0.6^*^	44 ± 0.5^*^	43 ± 0.6^*^	40 ± 0.6	<0.001
Creatinine, μmol/L	78 ± 3	80 ± 3	81 ± 3	79 ± 3	83 ± 3^*^	80 ± 2	0.024
Urea, mmol/L	4.5 ± 0.2	4.2 ± 0.1	4.2 ± 0.1	4.5 ± 0.1	5.4 ± 0.1^*^	5.2 ± 0.1^*^	<0.001
Fasting glucose, mmol/L	4.9 ± 0.1		5.1 ± 0.1	5.0 ± 0.1	4.8 ± 0.1	4.8 ± 0.1	0.001
Fasting insulin, μU/ml	4.96 ± 0.34		6.65 ± 0.62	6.01 ± 0.43	6.91 ± 0.44^*^	6.79 ± 0.48^*^	0.002
HOMA IR	1.08 ± 0.08		1.53 ± 0.16	1.34 ± 0.11	1.49 ± 0.11^*^	1.45 ± 0.11^*^	0.006
Leptin, ng/ml	2.15 ± 0.38		2.49 ± 0.45	2.36 ± 0.50	2.65 ± 0.52	2.19 ± 0.51	0.006
Cholesterol, mmol/L	4.6 ± 0.2			5.0 ± 0.2^*^	4.7 ± 0.2	4.4 ± 0.2	<0.001
HDL, mmol/L	1.13 ± 0.04			1.16 ± 0.04	1.17 ± 0.07	1.06 ± 0.04	0.097
LDL, mmol/L	2.93 ± 0.17			3.27 ± 0.21^*^	3.09 ± 0.20	2.85 ± 0.18	<0.001
Triglycerides, mmol/L	1.09 ± 0.11			1.16 ± 0.09	1.04 ± 0.07	1.08 ± 0.09	0.508
hs-CRP, mg/L	0.7 ± 0.3	0.4 ± 0.1	0.7 ± 0.3	1.2 ± 0.6	0.9 ± 0.4	0.7 ± 0.2	0.3
Aldosterone, ng/L	53 ± 9				75 ± 10	127 ± 27^*^	0.004
BNP, ng/L	8.3 ± 1.6				Undetectable^*^	Undetectable^*^	0.032
Renin, ng/L	19 ± 4				29 ± 4^*^	37 ± 4^*^	<0.001
RBC, 10^12^/L	4.96 ± 0.07					4.97 ± 0.07	0.9
Haematocrit, %	43.4 ± 0.4					43.1 ± 0.5	0.6
Hemoglobin, g/100 ml	14.6 ± 0.12					14.7 ± 0.18	0.6
MCV, fL	87.6 ± 1.1					86.9 ± 1.1^*^	0.001
MCHC, %	33.6 ± 0.12					34.0 ± 0.14^*^	0.002
TGMH, pg	29.4 ± 0.4					29.5 ± 0.4	0.3
WBC, 10^9^/L	5.52 ± 0.31					5.59 ± 0.29	0.7
Platelets, 10^9^/L	227.5 ± 12.8					221.8 ± 11.5	0.3
Reticulocytes, 10^9^/L	42.5 ± 3.3					42.7 ± 2.4	0.97
Total bilirubin, mg/l	8.2 ± 1.5					10.0 ± 2.4	0.19
Prothrombin time, %	91 ± 3					89 ± 3	0.3
Alkaline phosphatase, U/L	58 ± 5					57 ± 5	0.3
GGT, U/L	17 ± 1.5					17 ± 1.5	0.4
AST, U/L	27 ± 2					26 ± 2	0.6
ALT, U/L	29 ± 2					33 ± 4	0.2

Values are mean ± SEM;

* *p ≤ 0.05 vs. baseline*.

### Cardiovascular deconditioning

#### Orthostatic tolerance

Presyncopal LBNP-tilt test revealed a pronounced decrease in orthostatic tolerance with orthostatic tolerance time drop from 27 ± 1 min–on baseline to 9 ± 2 min–on R0. Before DI all subjects tolerated the first stage of test which consisted of an 80° tilt for 15 min. However, immediately after DI, 9 subjects out of 12 were unable to accomplish this tilt. Therefore, orthostatic tolerance time for non-finishers averaged at 4.95 ± 0.7 min. No significant correlation between percent decrease in plasma volume and post-immersion orthostatic tolerance time was found (Pearson *r* = −0.36; *p* = 0.28).

**Figure 3:**
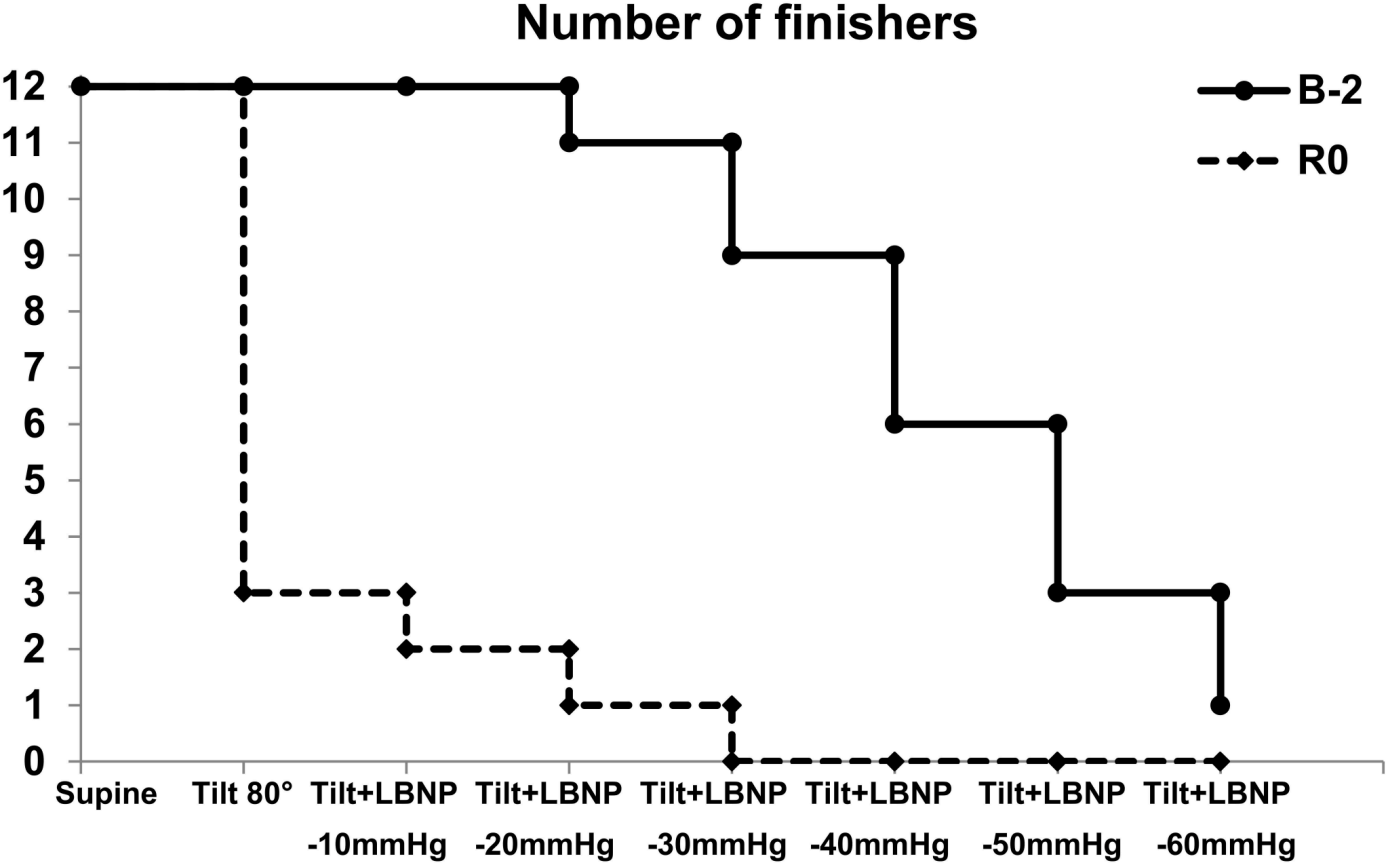
Number of finishers for different stages of LBNP-tilt test before and immediately after 3-day DI.

Tolerance for different LBNP steps is shown in Figure 3. Before DI, all subjects finished 10 mmHg LBNP step, a half tolerated 40 mmHg step, and one (subject E) accomplished the last step of 60 mmHg. After DI, only 2 of the 12 subjects finished the first LBNP step, and only 1 subject (subject E) finished the step of 20 mmHg. On R+1, all subjects finished the progressive tilt test.

#### Hemodynamic and autonomic responses to LBNP-tilt and progressive tilt

**Figure 4:**
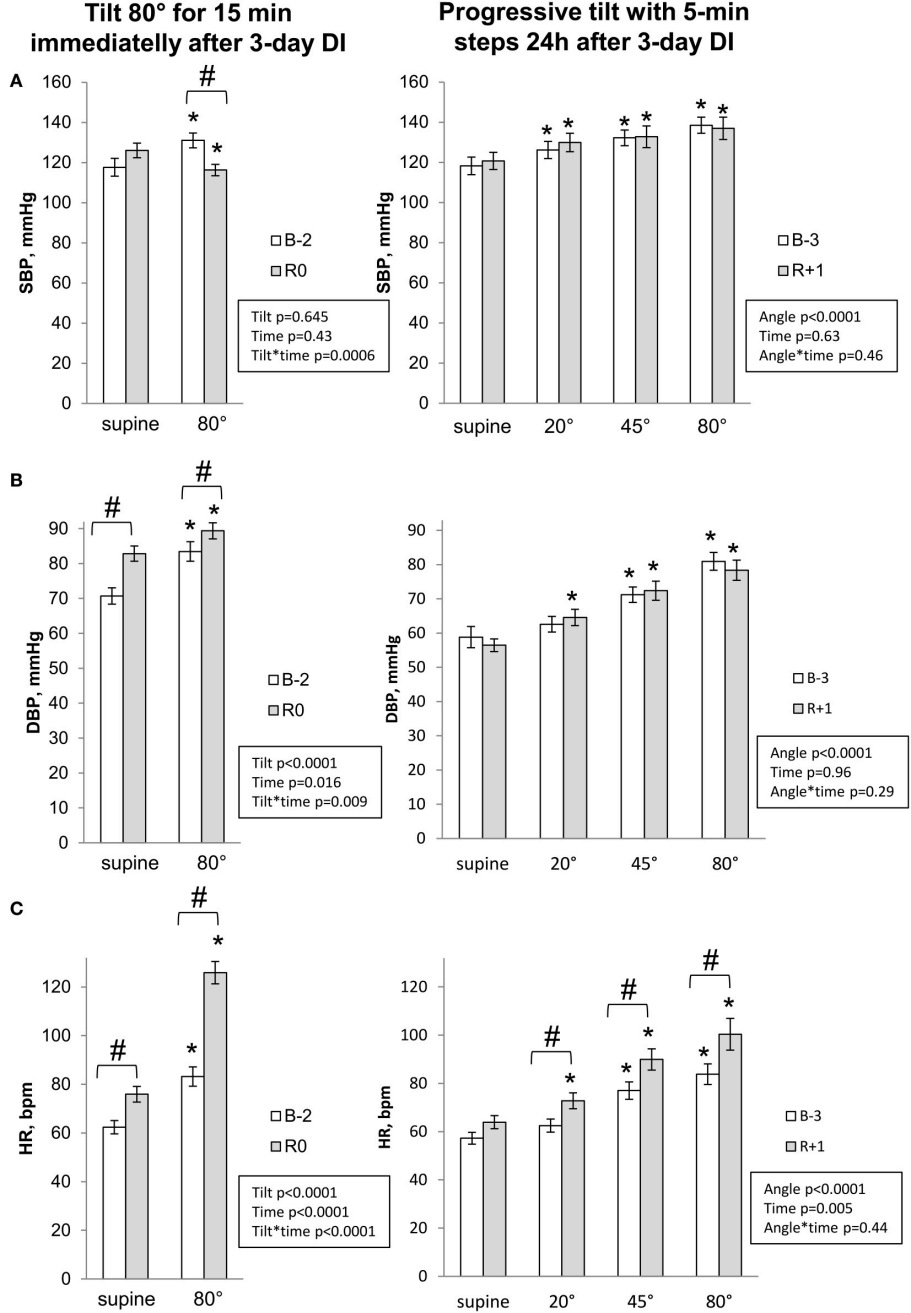
Cardiovascular changes during orthostatic tests for systolic **(A)** and diastolic **(B)** blood pressure and heart rate **(C)**. Data are mean ± SEM. ^*^p ≤ 0.05 vs. Supine; #*p* ≤ 0.05 vs. Before.

**Figure 5:**
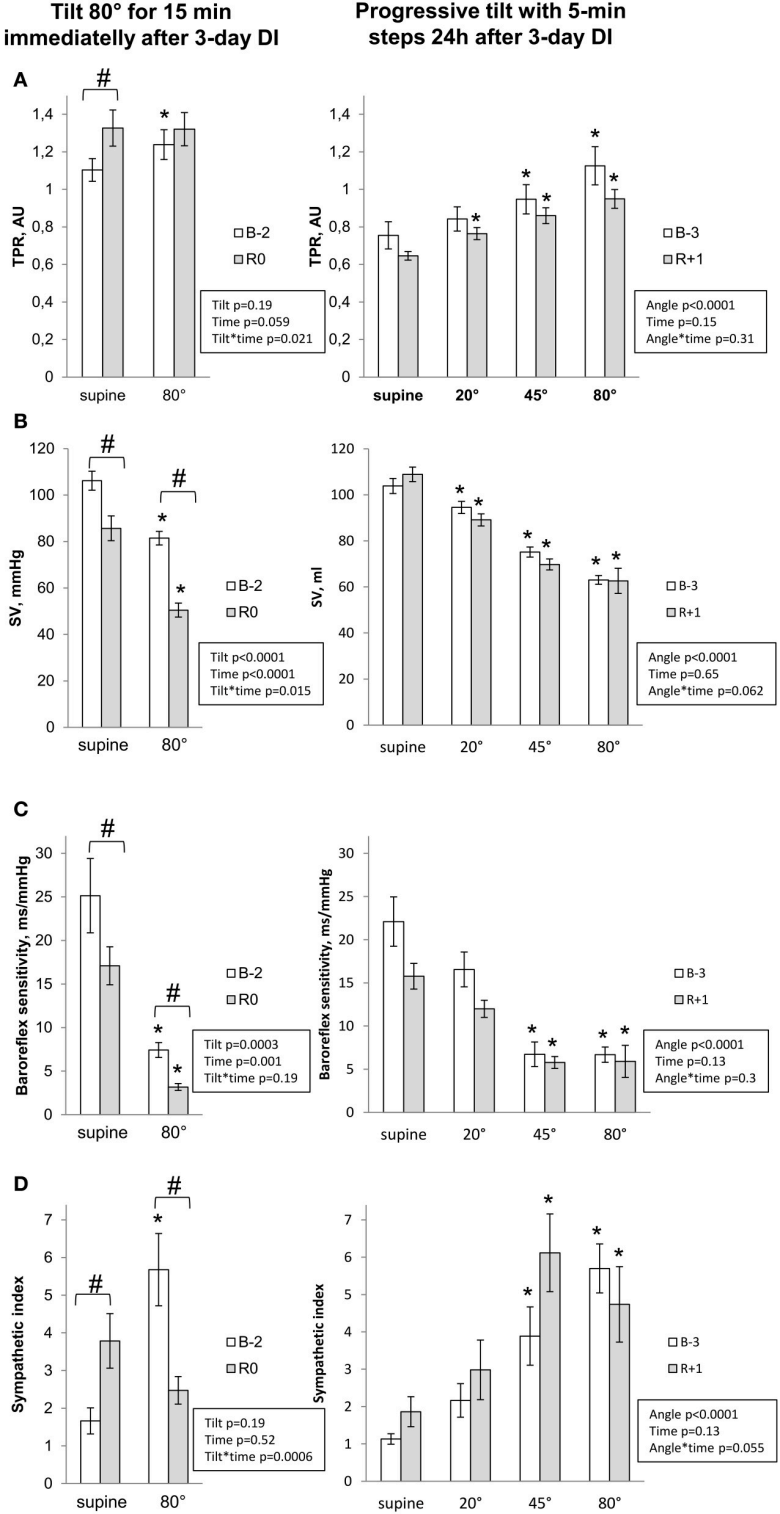
Cardiovascular changes during orthostatic tests for total peripheral resistance **(A)**, stroke volume **(B)**, spontaneous baroreflex sensitivity **(C)** and sympathetic index **(D)**. Data are mean ± SEM. ^*^p ≤ 0.05 vs. Supine; #*p* ≤ 0.05 vs. Before.

Hemodynamic and autonomic responses to LBNP-tilt and Progressive tilt tests are presented in Figures 4, 5. Pre-immersion supine baseline for diastolic blood pressure, HR, total peripheral resistance and sympathetic index was greater for LBNP-tilt compared to progressive tilt, most likely due to stress caused by using the LBNP equipment. Before DI, upright position provoked expected changes in central hemodynamics and cardiac autonomic neural control (increased blood pressure, HR, total peripheral resistance and sympathetic index; decreased stroke volume and baroreflex sensitivity). These changes were progressive with progressive tilt.

Post-immersion supine measurements on R0 showed significant increases in diastolic blood pressure, HR and total peripheral resistance, a 2-fold increase in sympathetic index, and a decrease in stroke volume and baroreflex sensitivity. On R+1, supine measurements did not differ significantly from pre-immersion levels.

Upright measurements showed a decrease in systolic blood pressure, stroke volume and baroreflex sensitivity, accompanied by pronounced tachycardia on R0. Total peripheral resistance and sympathetic index, which were already increased in supine, failed to further increase with orthostasis. On R+1, upright position was still accompanied by greater tachycardia. Interestingly, while tilt-induced sympathetic activation before DI increased progressively with verticalisation [reaching maximum at maximal angle of tilt (80°)], on R+1 the maximal sympathetic index was observed at 45° (difference between angles 45 and 80° before DI, *p* = 0.07; after DI, *p* = 0.8).

### Metabolism

#### Blood variables relevant to metabolism

All fasting blood variables relevant to metabolism remained within physiological limits (Table 2). DI increased insulin levels, while insulin resistance (HOMA-IR) increased by 43 ± 11% on R0 vs. baseline. Total cholesterol and LDL cholesterol fraction were moderately increased under immersion. Triglycerides and HDL remained nearly unchanged.

#### Oral glucose tolerance test

**Figure 6:**
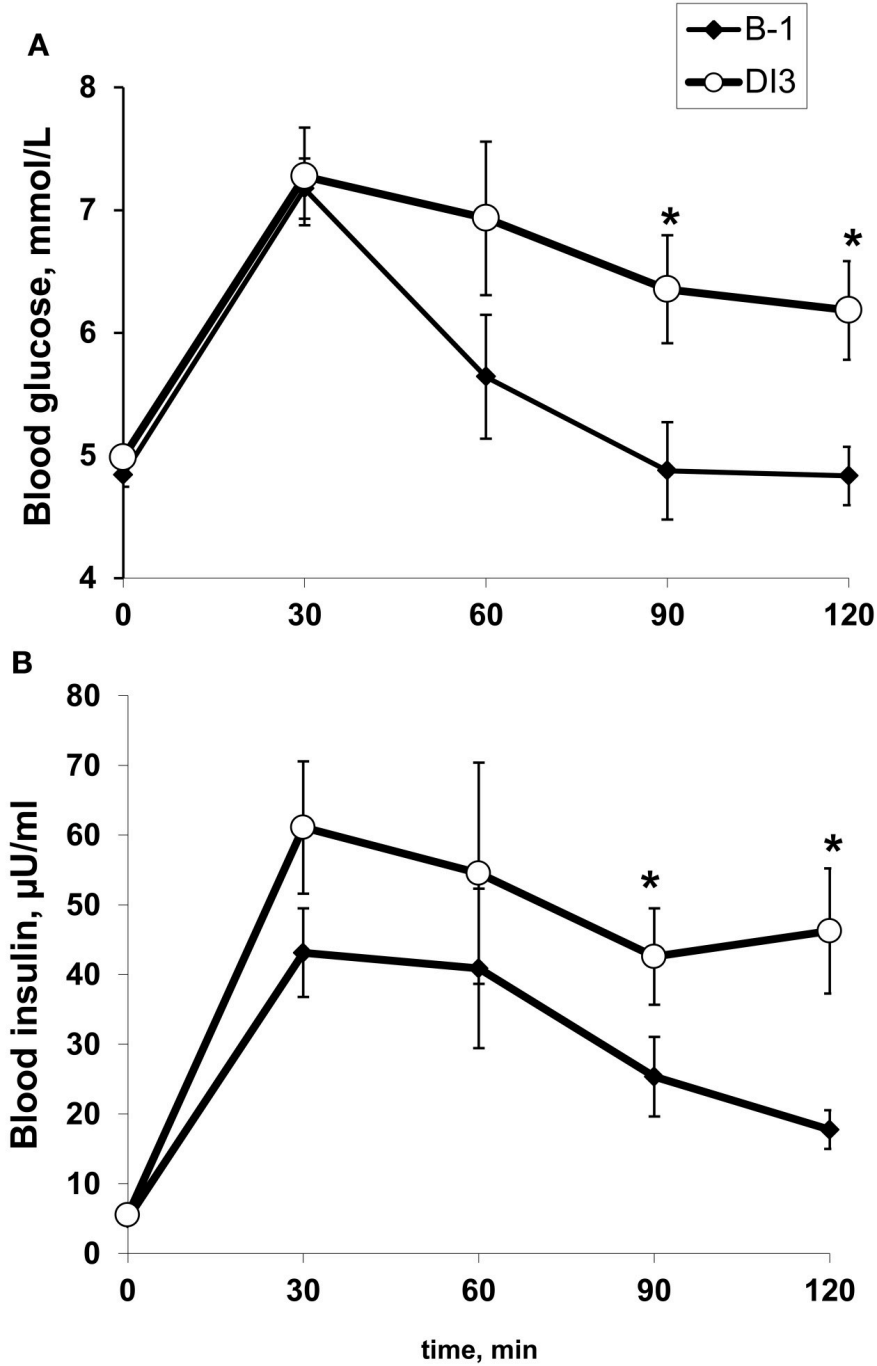
The effect of 48-h dry immersion on the glucose **(A)** and insulin **(B)** response to 75-g oral glucose tolerance test in 12 healthy subjects. Data are mean ± SEM. ^*^p ≤ 0.05 vs. B-1.

The response to oral glucose tolerance test is shown in Figure 6. Fasting glucose was beneath the pre-diabetic threshold (6.1 mmol/L) in all volunteers, and was not altered by DI. Glucose tolerance was compromised by DI in 11 subjects out of 12, and in 3 subjects this impaired glucose tolerance reached pre-diabetic level (more than 7.8 mmol/L 2 h after having a glucose drink). Net insulin response (AUC-Ins) increased by 72 ± 23% on DI3 compared to baseline. There was also a 14 ± 5% increase in the net glucose response (AUC-Glu), while incremental AUC for glucose response increased two-fold.

### Muscle tone

**Figure 7:**
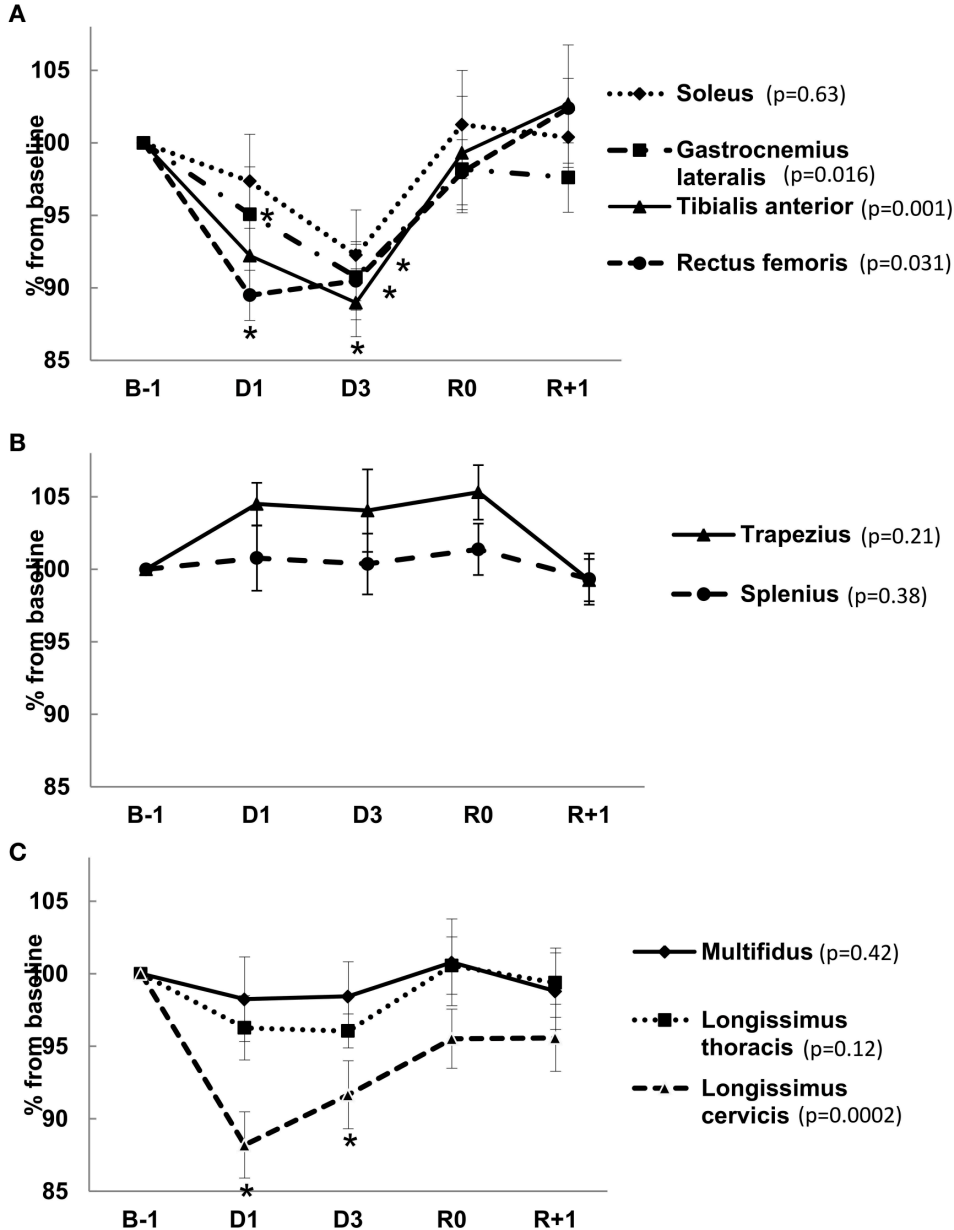
Percent change in muscle tone of leg muscles **(A)**, superficial neck and upper trunk muscles **(B)** and deep back muscles **(C)** 6 h following the onset (D1), on day 3 of immersion (D3), in recovery period on R0 (6 h following the end of DI) and on R+1. Data are mean ± SEM. ^*^p ≤ 0.05 vs. B-1.

Muscle tone variations under DI are shown in Figure 7. Global leg muscle tone, proxy measured by “frequency” parameter, was decreased by approximately 10% under immersion. This decrease was immediate (seen 6 h following the onset of DI) and especially pronounced for *m. rectus femoris* (results for our subjects are also outlined in Demangel et al., 2017). The tone of *m. gastrocnemius lateralis* significantly decreased on DI3. The tone of superficial muscles of the neck and upper trunk (*m. trapezius* and *m. splenius)* was not significantly modified under immersion. Behavior of deep back muscle tone had cervico-lombar gradient. Tone immediately dropped in the upper part (12% for *m. longissimus cervicis*), shifting to slight decrease on D3 in the middle (*m. longissimus thoracis*) and was not modified in the lower part (lumbar portion of *m. multifidus*). Six hours following the end of DI, muscle tone was completely restored.

### Day-night variations in urinary excretion

#### Urinary sodium

**Figure 8:**
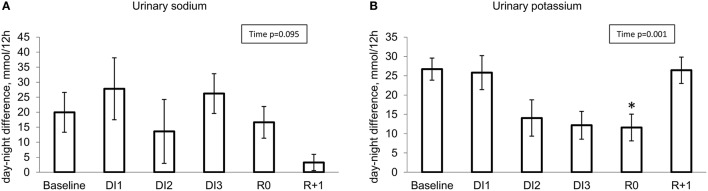
Day-night difference in renal excretion for sodium **(A)** and potassium **(B)**. ^*^p ≤ 0.05 vs. baseline.

At baseline, daytime excretion exceeded night-time excretion by approximately 20%. On DI3, day-night variations in Na^+^ excretion were preserved (Figure 8A).

#### Urinary potassium

At baseline, day-night differences in K^+^ excretion were clearly marked and stable, with 2.5 fold greater excretion in daytime. There was no effect on day-night difference in K^+^ excretion after DI1. At the start of DI2, night excretion tended to increase while day excretion tended to decrease, showing an important drop in day-night difference reaching significance on R0 (Figure 8B).

Interestingly, 24 h excretion was stable for K^+^ throughout the protocol, whereas 24 h Na^+^ excretion varied significantly within immersion, peaking at the onset of about 40% over baseline and drop at the end of DI of about 60% beneath baseline.

#### Urinary cortisol

**Figure 9:**
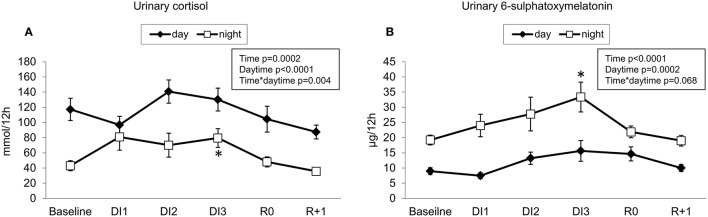
12-h renal excretion for cortisol **(A)** and melatonin metabolite **(B)**. ^*^p ≤ 0.05 vs. baseline.

Urinary cortisol had pronounced day-night oscillations. Cortisol levels tended to decrease in the first 10 h of DI, then to increase on the night of DI1, however, these changes were within physiological limits. Day-night differences in cortisol excretion were smoothed at the onset of immersion, but restored on DI2. Cortisol level recovered immediately after DI (Figure 9A).

#### Urinary aMT6s

Urinary aMT6s had pronounced day-night oscillations, which tended to accentuate during immersion mainly due to the increased night release. On R0, day-night differences in aMT6s secretion were diminished, suggesting readaptation to normal conditions (Figure 9B).

### General discomfort and pain during DI

In general, the majority of subjects described the discomfort level experienced under DI as 30–45 out of 100. Some subjects reported pronounced discomfort at nighttime. We observed important inter-subject variance in auto-reported discomfort level, varying from 0 to 88. There was no significant difference in discomfort level between the different days of DI. Eleven out of the 12 subjects reported moderate back pain under DI, which was predominantly localized in the lumbar area for 10 subjects. Lumbar pain intensity as estimated on DI2 was 3.8 ± 0.7 on the scale of 1–10. There was a positive correlation between lumbar pain intensity and mean 24-h urinary cortisol during DI (199 ± 24 mmol/24 h) (Pearson *r* = 0.64; *p* = 0.02). As well, we observed a positive correlation between mean discomfort level and mean 24-h urinary melatonin metabolite under DI (40.5 ± 5.4 μg/24 h) (Pearson *r* = 0.67; *p* = 0.018).

### Blood electrolytes, blood count, liver-related biochemistry, hs-CRP

Data are detailed in Table 2. Plasma electrolytes showed modest changes without clinical relevance. Blood count remained unchanged except for a modest but highly significant decrease in RBC volume, accompanied by slight increase in MCHC on R+1 vs. B-3. Blood GGT, ALAT, ASAT, alkaline phosphatase, total bilirubin, prothrombin time and serum albumin were not modified on R+1 vs. B-3. CRP levels were unaltered by DI.

## Discussion

### Main findings

Orthostatic tolerance time dropped from 27 ± 1 min to 9 ± 2 min after DI. Impairment in glucose tolerance was significantly pronounced. Net insulin response increased by 72 ± 23% on DI3 compared to baseline. Global leg muscle tone was reduced by approximately 10% with the DI protocol. Day-night changes in temperature, heart rate and blood pressure were preserved on the third day of DI. Day-night levels of urinary K^+^ were reduced, beginning on the second day of immersion, while 24-h K^+^ excretion remained stable. Urinary cortisol and melatonin metabolite levels increased with DI, although within normal limits. A positive correlation between lumbar pain intensity estimated on DI2 and mean 24-h urinary cortisol under DI was observed.

### Need for strict DI without daily rise

In the present study, we observed pronounced cardiovascular deconditioning after 3 days of strict DI. This deconditioning was characterized by a predisposition to orthostatic intolerance and tachycardia.

#### Orthostatic intolerance

Seventy-five percent of our subjects were intolerant to orthostasis on R0 after 3 days of strict DI. In comparison with literature data using chi-square test, orthostatic intolerance rate after strict bed rest without countermeasures does not differ significantly from our findings: 5/11 (45%)-after 4 days (*p* = 0.15), 4/6 (67%)-after 14 days (*p* = 0.7), 5/9 (56%)-after 28, or 30 days (*p* = 0.35), and 4/7 (57%)-after 42 days of HDBR (*p* = 0.4) (Pavy-Le Traon et al., 1999).

However, the reported rate of orthostatic intolerance following non-strict DI seems much less-approximately 15 to 40% of subjects for 1 to 7 days of DI. Indeed, after 24 h of DI, 2 subjects out of 10 were reported to be intolerant to the orthostatic test on R0 (*p* = 0.01), after 1.5 days of DI–1 out of 6 (*p* = 0.02) (Navasiolava et al., 2011a), after 3 days of DI–1 out of 6 (*p* = 0.02) (Iwase et al., 2000) and 0 out of 4 (*p* = 0.009) (Miwa et al., 1997), after 5-day DI–4 out of 7 (*p* = 0.4) (one was already intolerant before DI) (Coupé et al., 2013), and after 7 days of DI–2 out of 6 (*p* = 0.09) (Iarullin et al., 1987) and 1 out of 8 (*p* = 0.006) (Navasiolava et al., 2011b). The main differentiator of our DI was the maintenance of supine position throughout. Our data suggest that daily short periods out of the water tank in sitting or standing positions represent a powerful countermeasure against orthostatic intolerance induced by DI. Periodic short gravitational stimuli appear to be effective countermeasures, maintaining gravitational tolerance. Vernikos et al. (1996) demonstrated a very efficient preventive effect of a short period in a standing position (for 2 h daily) against orthostatic intolerance after 4 days of HDBR. The beneficial effect of LBNP for 20 min/day during both spaceflight and bed rest was discussed in a review by Clement and Pavy-Le Traon (2004). They cite the evidence that daily centrifugation for 30–45 min reduces most of the physiological markers associated with orthostatic intolerance. Thus, a daily short period of orthostatic stress is sufficient to reduce the risk of orthostatic intolerance after simulated weightlessness. Daily short term gravitational load might also promote the preservation of baroreflex sensitivity and autonomic balance.

#### Tachycardia

Resting tachycardia measurements observed immediately after immersion at R0 suggest that even the supine position out of water represents an additional workload for subjects exposed to immersion. Strict DI induces more pronounced cardiovascular deconditioning than non-strict DI, even if daily rise is very short. Similar to our finding, an increase in both asleep and awake HR was documented following 5-to-10-day space mission (Fritsch-Yelle et al., 1996). The majority of previous DI studies did not report significant changes in resting supine HR following DI (Navasiolava et al., 2011a). Tachycardia, when measured in an upright position, demonstrated a 50% increase at the first rise following DI (125 ± 5 bpm vs. 83 ± 4 bpm). Previous studies reported a 25% increase in upright HR after 3-day DI (Iwase et al., 2000), 30%- after 5-day DI (Coupé et al., 2013), 40% and 48%- after 7-day DI (Iarullin et al., 1987; Navasiolava et al., 2011a), and 50%- after 10-day DI (Panferova, 1976). Taken together, literature suggests that resting and upright tachycardia readings are emphasized and enhanced following strict DI compared to non-strict DI protocols.

#### Sympathetic regulation of cardiovascular functions

Sympathetic neural control is extremely important in maintaining blood pressure homeostasis against gravity (Mano, 2005). In our study, strict DI clearly affected cardiac sympathetic neural control and baroreflex sensitivity. Supine sympathetic index, which presumably reflects resting cardiac sympathetic activity, was more than 2-fold increased immediately after DI, suggesting an activation of sympathetic nervous system. Supine sympathetic index failed to further increase in response to tilt, unlike previous DI studies, such as that by Miwa et al. (1997), who found an increase in upright sympathetic index following 3-day DI. Observed alteration in the sympathetic index response to progressive tilt may be related to dysregulation of high–low pressure baroreceptors. Therefore, a decrease in sympathetic index may represent a factor in orthostatic intolerance. We found that upright sympathetic index on R0 tended to correlate with orthostatic tolerance time (Pearson *r* = 0.54; *p* = 0.068).

#### Vasoconstriction to orthostasis

Total peripheral resistance is an integrative characteristic of overall resistance of peripheral vasculature in the systemic circulation. Insufficient vasoconstriction (increase in total peripheral resistance) to orthostatic stimuli is an acknowledged major factor for microgravity-induced orthostatic hypotension (Zhang, 2001). Astronauts intolerant to orthostatism fail to adequately increase TPR when upright (Buckey et al., 1996). Increased resting total peripheral resistance observed in our study suggests greater basal vasoconstriction and therefore a decrease in the reserve of vasoconstriction, with limitation of the vasoconstrictive response. According to Convertino hypothesis (Convertino, 1999), diminished vasoconstrictive reserve may be the main mechanism of vasoconstrictor insufficiency in cases of orthostatic intolerance. The maximal capacity of vasoconstriction is not altered under microgravity (Convertino, 1999), but hypovolemia might induce an increase in baseline vasoconstriction and thus decrease the vasoconstrictive reserve. However we did not observe direct correlation between upright total peripheral resistance at R0 and orthostatic tolerance time (Pearson *r* = 0.47; *p* = 0.12), suggesting involvement of additional factors into orthostatic intolerance.

#### Water-sodium balance and plasma volume

Resetting of water-sodium balance under DI, accompanied by acute BNP increase and renin-aldosterone suppression, usually occurs over the first to second day, with a subsequent return in hormonal concentrations to their usual level (Leach Huntoon et al., 1998; Navasiolava et al., 2011a,c; Coupé et al., 2013). We did not assess the initial hormonal responses. The observed moderate rise in renin at the end of DI may be related to the slight decrease in dietary sodium under DI. However we did not find direct correlation between the decrease in dietary sodium and the increase in renin at the end of DI (Pearson *r* = −0.36; *p* = 0.26). Similarly, Shulzhenko et al. (1980) reported a minor increase in plasmatic renin on DI7 and in aldosterone on DI5 and DI6. The observed decrease in plasma volume of 16% is in accordance with the data described in other DI studies, with 15–20% hypovolemia (Leach Huntoon et al., 1998; Navasiolava et al., 2011a; Coupé et al., 2013). The similarity in hypovolemia numbers obtained by the CO technique and the Dill and Costill estimation suggests the accuracy of the indirect method of plasma volume estimation by Hb and Hct count under DI. The observed greater reduction in plasma volume in fitter subjects was in line with Convertino (1996a).

#### What may be responsible for this decrease in orthostatic tolerance?

Hypovolemia appears to be the major contributor for the observed rapid cardiovascular impairment in DI. However, the degree of hypovolemia in our study is similar to that reported for non-strict DI (15%–Leach Huntoon et al., 1998; 17-18%–Coupé et al., 2013; 15%–Navasiolava et al., 2011c; 12-16%–Gogolev et al., 1980). Moreover, the percentage decrease in plasma volume did not directly correlate with orthostatic tolerance time (Pearson *r* = −0.36; *p* = 0.28). The degree of hypovolemia develops within the first 24 h and is not greatly dependant on the duration of actual or simulated microgravity, whereas the degree of orthostatic intolerance tends to increase with extension of microgravity. Aside from a reduction in plasma volume, other mechanisms including increased lower limb venous capacitance (Convertino, 1996b), leg muscle tone diminution, compromised sympathetic regulation (Convertino, 2002; Mano, 2005), myocardial function and baroreflex sensitivity (Engelke et al., 1996; Convertino, 2002) may also contribute to cardiovascular deconditioning following DI. Vascular impairment at macro- (Zhang, 2001) and microvascular (Coupé et al., 2009) level promoted by physical inactivity and reduced shear stress (de Groot et al., 2006), in addition to metabolic, hormonal and vestibular changes may also play a role.

### Rapid and profound metabolic changes induced by strict DI

The negative metabolic effects of DI are mainly related to increased inactivity. DI rapidly impaired glucose metabolism and lipid profile, inducing a decrease in insulin sensitivity and dyslipidemia. The same changes were observed in bed rest experiments (Blanc et al., 2000; Hamburg et al., 2007; Bergouignan et al., 2010). Even short-time physical inactivity appears sufficient to impair metabolism (Hamburg et al., 2007; Coupé et al., 2013; this study).

Some studies suggest that fasting glucose is not modified by DI, and fasting insulin is somewhat increased following 7-day DI (Navasiolava et al., 2011a). However, the effect of DI on glucose tolerance has not previously been explicitly investigated. This study is believed to be the first to examine the effect of DI on oral glucose tolerance test in depth. Data in the published literature indicate that bedrest (BR) increases insulin response to glucose loading without deterioration in glucose tolerance (Kiilerich et al., 2011–non-strict 7-day BR in men with allowed sitting 5h/day, Blanc et al., 2000–7-day strict HDBR in women, Dirks et al., 2016–7-day strict BR in men, mixed-meal tolerance test) or with slight increase in the net glucose response of about 6% (Hamburg et al., 2007–5-day BR in men and women, Heer et al., 2014–21-day BR in men). Globally, in BR, an increased insulin response is able to prevent glucose increase, or at a minimum, this glucose increase remains relatively moderate. In our DI experiment, a shorter exposure (2 days) seems to induce a greater impairment in glucose tolerance (14%-increase in the net glucose response). However, caution should be employed when interpreting these results as our subjects showed pre-immersion response to oral glucose intake with a faster decline in glucose concentration compared to baseline of bedrest studies reported by Heer et al. (2014) and by Hamburg et al. (2007), whereas post-immersion response was much closer to that observed after those bedrest protocols. This difference in baseline measurements may be related to individual sensitivity of subjects and limits the possibility of direct comparison.

Increased blood glucose and dyslipidemia may compromise endothelial integrity and microvascular functions (Hamburg et al., 2007; Yuan et al., 2015), thus indirectly contributing to orthostatic intolerance. However, we did not observe any direct correlation between induced glycemia on DI3 and orthostatic tolerance time following DI (Pearson *r* = −0.32; *p* = 0.31).

### Day-night variations

Day-night variability in HR, blood pressure and body temperature on DI3 did not differ from baseline, suggesting a good adaptation to DI conditions. Circadian rhythm of K^+^ excretion is one of the most consistent and stable physiological circadian fluctuations (Gumz et al., 2015). It is independent of cyclic potassium intake, adrenal hormones, changes in plasma potassium, renal nerves and sodium cyclic excretion. Physiologic circadian rhythm in potassium excretion has high amplitude and is driven by a brain oscillator. As suggested by Gumz and Rabinowitz (2013), fluctuations in potassium excretion are regulated by circadian fluctuations in K^+^ transporter gene expression. “Predictive” signals from central nervous system (CNS) cyclically change kidney sensitivity for the excretory commands, thus preparing increase in renal excretion for the period of expected potassium intake with meal (Gumz et al., 2015).

In our study, circadian rhythm in potassium excretion was preserved, however we observed a delayed diminution in its amplitude. A possible rationale is that DI might induce a forward shift of day-night cycle (under DI the subjects have sleeping difficulties and might fall asleep later). Consequently, the 19:00–07:00 urine pool would contain more “daily” urine than before, so night kaliuresis would appear to be elevated. However, night melatonin was not decreased as expected in case of increased “contamination” of night urine with daily portion. This suggests another potential explanation—a certain disturbance in circadian regulation itself. The observed delayed changes in renal K^+^ excretion variance (and delayed restoration) might reflect a change in its “predictive”/feedforward regulation by homeostatic system of CNS (Gumz et al., 2015). Other studies have also shown that circadian rhythms could be modified by spaceflight (Fuller et al., 1994; Guo et al., 2014) and bed rest (Millet et al., 2001; Pavy-Le Traon et al., 2007; Liang et al., 2012). Bed rest did not change the excretion of K^+^, but altered the rhythmicity and circadian amplitude of Na^+^ excretion (Millet et al., 2001; Liang et al., 2012).

### Stress is moderate in DI and might be related to back pain

A relatively modest increase in cortisol suggests quite a moderate physiological stress effect of DI. During immersion, the increase in urinary cortisol level was more evident at night. It may be related to the greater perception of the stress factors inherent to DI at night time. Interestingly, in the first hours of immersion, a drop in cortisol levels was observed. In part, this is likely due to the fact that short time immersion is quite comfortable and relaxing, which is why the method is employed in a spa setting (Navasiolava et al., 2011a). The positive correlation between the change in urinary cortisol and self-reported lumbar pain level confirms the role of cortisol in response to stress under DI.

This lumbar pain was not accompanied by muscle spasm. Indeed, an increase in lower back muscle tone might be expected, as lumbar pain should induce a reflex contraction of the lumbar paravertebral muscles. However, direct support withdrawal immediately switches off the tone of postural muscles (Navasiolava et al., 2011a). We observed this immediate response for antigravity leg muscles and upper deep back, but not for lower deep back. Muscle tone stability in lower deep back could represent an averaged effect of immersion (which decreases the tone) and back pain (which increases tone). The deep muscles of the lower back, which play an important role in stabilizing the joints within the spine, may preserve their tone to counteract lumbar deformation associated with back pain.

Night increase in melatonin might aim to counteract the effect of unusual environment and promote sleeping. Positive correlation between urinary melatonin and global discomfort level under DI suggests the “efforts” of melatonin to counteract discomfort.

## Conclusion

Dry immersion (DI) induces an accelerated model of cardiovascular deconditioning in response to microgravity. The “support unloading” induced by DI provides rapid and profound cardiovascular deconditioning. This is exemplified by increased plasma volume loss, orthostatic intolerance, pronounced autonomic changes, pronounced metabolic impairment, rapid and profound decreases in muscle tone, and influence on circadian rhythms. DI is tolerated well enough despite backache, and shows rather moderate stress effect. Such rapid, profound and quickly reversible gravitational deconditioning renders the strict DI model extremely significant to test countermeasures for microgravity-induced deconditioning and physical inactivity-related pathologies.

## Author contributions

Conception and design of the study: MC, NN, GG, and RM. Data acquisition and sample analysis: SD, LA, RM, VR, FL, LT, and RW. Analysis and interpretation of results, drafting and revising the article: All authors.

### Conflict of interest statement

The authors declare that the research was conducted in the absence of any commercial or financial relationships that could be construed as a potential conflict of interest.

## Abbreviations

ALT: Alanine Aminotransferase
aMT6s: 6-sulphatoxymelatonin
ANOVA: analysis of variance
AST: Aspartate Aminotransferase
AUC: area under curve
B: baseline
BMI: body mass index
BNP: blood natriuretic peptide
BR: bed rest
DI: dry immersion
DPV (%): plasma volume percent change
GGT: Gamma-Glutamyl Transferase
Hb: hemoglobin
Hct: hematocrit
HDBR: head down bed rest
HOMA-IR: homeostasis model assessment-insulin resistance index
HR: heart rate
hs-CRP: high-sensitivity C-reactive protein
LBNP: lower body negative pressure
MCHC: mean corpuscular hemoglobin concentration
MCV: mean corpuscular volume
R: recovery
RBC: red blood cells
SV: stroke volume
${\dot{\text{V}}\text{O}}_{2}$max: maximal oxygen uptake.
