# Supplementary material for: Multi-System Deconditioning in 3-Day Dry Immersion without Daily Raise
Source: Front Physiol. 2017 Oct 13;8:799. doi: 10.3389/fphys.2017.00799 (PMC5645726; doi:10.3389/fphys.2017.00799)
Supplement: Supplementary file 1 [file Table1.DOCX]

**Table S1. Nutrition in dry immersion study**

Values are mean±SEM; *p≤0.05 vs. B-1

|  | **B-3** | **B-2** | **B-1** | **DI1** | **DI2** | **DI3** | **R0** | **R+1** | **_p value_** |
| --- | --- | --- | --- | --- | --- | --- | --- | --- | --- |
| Energy (kcal) | 2816±41 | 2825±40 | 2825±40 | 2277±30* | 2267±48* | 2268±46* | 2819±42 | 2825±41 | <0.001 |
| Proteins (g) | 86±2 | 88±2 | 86±2 | 79±2* | 83±2 | 82±2* | 86±2 | 85±2 | <0.001 |
| Fats (g) | 112±2* | 113±1* | 106±2 | 93±1* | 89±2* | 89±2* | 112±2 | 113±2* | <0.001 |
| Carbohydrates (g) | 357±5* | 355±7* | 377±6 | 272±4* | 276±6* | 277±5* | 360±6* | 357±5* | <0.001 |
| Sodium (g) | 4.30±0.09* | 3.29±0.08 | 3.38±0.06 | 2.30±0.03* | 3.81±0.12* | 2.86±0.09* | 3.55±0.06* | 2.85±0.04* | <0.001 |
| Chlorine (g) | 4.52±0.09* | 3.66±0.15* | 3.26±0.11 | 3.41±0.05 | 3.62±0.09 | 2.98±0.07 | 3.65±0.11* | 4.44±0.07* | <0.001 |
| Potassium (g) | 3.97±0.05 | 4.16±0.04 | 3.78±0.08 | 3.63±0.05 | 4.16±0.05* | 2.29±0.05* | 3.76±0.05 | 4.10±0.05 | <0.001 |
| Calcium (mg) | 1043±9* | 953±10* | 1169±15 | 914±7* | 926±17* | 1020±25* | 1168±26 | 1022±13* | <0.001 |
| Phosphorus (mg) | 1267±21* | 999±16* | 1546±28 | 1267±17* | 1091±28* | 1073±27* | 1426±26* | 1469±27 | <0.001 |
