# Supplementary material for: Multi-System Deconditioning in 3-Day Dry Immersion without Daily Raise
Source: Front Physiol. 2017 Oct 13;8:799. doi: 10.3389/fphys.2017.00799 (PMC5645726; doi:10.3389/fphys.2017.00799)
Supplement: Supplementary file 2 [file Table2.PDF]

Table S2. Correlations

Correlation	Pearson r	p
**Orthostatic intolerance**		
OTT R0 – DPV%	-0.357	0.28
OTT R0 – SI_R0_end tilt	0.543	0.068
OTT R0 – height	0.508	0.092
OTT R0 – $\dot{V}O_2max$ _baseline	0.340	0.279
OTT R0 – PV baseline	0.356	0.256
OTT R0 – OGTT_2h_glucose_DI3	-0.320	0.311
OTT R0 – BMI_baseline	-0.276	0.385
OTT R0 – lumbar pain DI2	0.214	0.505
OTT R0 – mean discomfort during DI	0.405	0.191
**Fitness and plasma volume**		
DPV% – VO_2max_baseline	-0.764**	0.006
Relative PV(ml/kg) baseline – $\dot{V}O_2max$ _baseline	0.67*	0.017
**Glucose intolerance/metabolic impairment**		
OGTT_2h_glucose_DI3 – lumbar pain DI2	-0.078	0.809
OGTT_2h_glucose_DI3 – $\dot{V}O_2max$_baseline	-0.590*	0.043
OGTT_2h_glucose_baseline – $\dot{V}O_2max$_baseline	-0.356	0.256
OGTT_2h_glucose_DI3 – UFC 24h_mean during DI	0.054	0.868
**Pain and discomfort**		
Lumbar pain_DI2 – UFC 24h_mean during DI	0.643*	0.024
Lumbar pain DI2 – OTT R0	0.214	0.505
**Cortisol and glucose**		
UFC 24h_mean during DI – Fasting glucose DI3	0.000	0.999
UFC 24h_mean during DI – OGTT_2h_glucose_DI3	0.054	0.868
**Cortisol and sympathovagal balance**		
UFC 24h_mean during DI – SI_R0_supine	-0.355	0.258
UFC 24h_mean during DI – SI_R0_end tilt	-0.070	0.828
**Other**		
Relative PV(ml/kg) baseline – DPV%	-0.320	0.337
SI_R0_end tilt – height	0.575	0.051
Renin change on R0 – Dietary sodium change on DI3	-0.355	0.257
**Melatonin**		
Melatonin 24h_mean during DI – lumbar pain DI2	0.521	0.082
Melatonin 24h_mean during DI – mean discomfort score DI1-DI3	0.665*	0.018
Melatonin 24h_mean during DI – OTT R0	0.707*	0.010
Melatonin 24h_mean during DI – UFC 24h_mean during DI	0.273	0.391
UFC 24h_mean during DI – OTT R0	0.188	0.558
**TPR**		
TPR_R0_end tilt – OTT R0	0.472	0.121
TPR_R0_end tilt – SI_R0_end tilt	0.178	0.579
**Muscle tone**		
Percent change in *m. rectus femoris* tone _DI3 – OTT R0	0.270	0.483
Percent change in *m. rectus femoris* tone_DI3 – TPR_R0_end tilt	0.110	0.779

OTT - orthostatic tolerance time; R0 – first day of recovery; DPV% - plasma volume percent change; SI - sympathetic index; $\dot{V}O_2max$ - maximal oxygen uptake; PV - plasma volume; OGTT - oral glucose tolerance test; DI1, DI2, DI3 - first, second and third days of dry immersion; BMI - body mass index; DI - dry immersion; UFC - urinary free cortisol; TPR – total peripheral resistance.
